# Salinity Stress Does Not Affect Root Uptake, Dissemination and Persistence of *Salmonella* in Sweet-basil (*Ocimum basilicum*)

**DOI:** 10.3389/fpls.2017.00675

**Published:** 2017-05-02

**Authors:** Nirit Bernstein, Shlomo Sela (Saldinger), Nativ Dudai, Elena Gorbatsevich

**Affiliations:** ^1^Institute of Soil, Water and Environmental Sciences, Agricultural Research Organization, Volcani CenterRishon LeZiyyon, Israel; ^2^Department of Food Quality and Safety, Agricultural Research Organization, Volcani CenterRishon LeZiyyon, Israel; ^3^Unit of Medicinal and Aromatic Plants, Newe Ya’ar Research Center, Agriculture Research OrganizationRamat Yishay, Israel

**Keywords:** contamination, basil, internalization, human pathogens, persistence, root, salt stress, *Salmonella*

## Abstract

Crop produce can be contaminated in the field during cultivation by bacterial human pathogens originating from contaminated soil or irrigation water. The bacterial pathogens interact with the plant, can penetrate the plant via the root system and translocate and survive in above-ground tissues. The present study is first to investigate effects of an abiotic stress, salinity, on the interaction of plants with a bacterial human pathogen. The main sources of human bacterial contamination of plants are manures and marginal irrigation waters such as treated or un-treated wastewater. These are often saline and induce morphological, chemical and physiological changes in plants that might affect the interaction between the pathogens and the plant and thereby the potential for plant contamination. This research studied effects of salinity on the internalization of the bacterial human pathogen *Salmonella enterica* serovar Newport via the root system of sweet-basil plants, dissemination of the bacteria in the plant, and kinetics of survival *in planta*. Irrigation with 30 mM NaCl-salinity induced typical salt-stress effects on the plant: growth was reduced, Na and Cl concentrations increased, K and Ca concentrations reduced, osmotic potential and anti-oxidative activity were increased by 30%, stomatal conductance was reduced, and concentrations of essential-oils in the plants increased by 26%. Despite these physical, chemical and morphological changes in the plants, root internalization of the bacteria and its translocation to the shoot were not affected, and neither was the die-off rate of *Salmonella in planta*. The results demonstrate that the salinity-induced changes in the sweet-basil plants did not affect the interaction between *Salmonella* and the plant and thereby the potential for crop contamination.

## Introduction

It is now recognized that human pathogens from soil or irrigation water may become associated with the plant following external contamination (Reviewed by Erickson, 2012), penetrate internal plant tissues via the shoot as well as the root, and translocate and survive inside the plant (Guo et al., 2002; Solomon et al., 2002; Jablasone et al., 2005; Bernstein et al., 2007b; Klerks et al., 2007a; Mootian et al., 2009; reviewed by Erickson, 2012; Hirneisen et al., 2012; Kisluk and Yaron, 2012). The interaction between the plants and human pathogens is not well understood and due to potential health risks involved, is currently the focus of numerous investigations (Barak and Schroeder, 2012; Wiedemann et al., 2015). The physiological and molecular responses of the plant to colonization with bacterial human pathogens and the factors which affect it are the focus of numerous studies (Brandl, 2006; Klerks et al., 2007b; Schikora et al., 2008; Teplitski et al., 2009; Gu et al., 2011; Gutierrez-Rodriguez et al., 2012; Hirneisen et al., 2012; Markland and Kniel, 2015).

A common source for contamination of agricultural fields by human pathogenic bacteria are soil amendments such as organic manures and marginal irrigation waters such as treated or un-treated effluents. These are often saline and contain higher concentrations of Na and Cl than tap water, as well as higher levels of microorganisms including human pathogenic bacteria (Gerba and Smith, 2005; World Health Organization [WHO], 2006; Scheierling et al., 2011). Their application to the field may therefore impose salt-stress on the plants and increase health risks involved in crop contamination with human pathogenic microorganisms.

Salt-stress induces a range of molecular, physiological and morphological changes in plant tissues, which in addition to their effect on the performance of the plant may affect its response to contaminating human pathogens. Specifically, salinity increases root branching (Bernstein and Kafkafi, 2002) and hence the openings potentially available for bacterial internalization through the root; it stimulates early root vasculature development and hence the potential for root-shoot transport of root internalized pathogens; salinity-induced changes in quantity of root exudates and concentration of specific exudate constituents such as free amino acids (Marin et al., 2010) may affect pathogen chemotaxis toward roots (Klerks et al., 2007b); and inhibited root elongation reduces the volume of soil explored by roots and hence its contact with pathogens (Reviewed by Bernstein and Kafkafi, 2002; Bernstein, 2013). Additionally, increased osmotic potential and salts concentrations in the plant cells under salinity, and intensified oxidative or anti-oxidative responses (Hasegawa et al., 2000; Munns and Tester, 2008; Bernstein et al., 2010) may affect *in planta* survival of internalized bacteria. Furthermore, exposure to abiotic stresses such as salinity, can prime various antibacterial induced resistance phenomena in plants (Beckers and Conrath, 2007; Foyer et al., 2016) which may affect human pathogens. Plants are known to accumulate antimicrobial compounds, i.e., phytoalexins, as a result of infection or stress. These elicitators include a wide range of chemical compounds of microbial and plant origin, as well as inorganic salts that typically accumulate in the plant under salinity (reviewed in Kuc, 1995; Boller and Felix, 2009). In basil, salinity increases the accumulation of salts in the plant and augment the concentration of essential oils, which are known to have antibacterial properties (Bernstein et al., 2009b). Therefore, among other effects, salinity may prime systemic resistance in plants, which might influence the plant’s response to microorganisms.

Although *Salmonella* is known to be tolerant to high NaCl levels (Kang et al., 2007), no information is available concerning effects of salinity on the association of human pathogenic bacteria with plants. Such data is important for understanding the potential for contamination of produce by pathogens from contaminated water or manure under saline conditions.

*Salmonella* is a common etiological agent of food-borne infections (Sivapalasingam et al., 2004), and Salmonellosis outbreaks have been reported to be associated with the consumption of numerous plant products (Campbell et al., 2001; Horby et al., 2003; Fatica and Schneider, 2011) and recently also with sweet basil (Pakalniskiene et al., 2006; Pezzoli et al., 2007). *Salmonella* from contaminated soil can internalize sweet-basil through the root, and transport *in planta* to vegetative and reproductive organs of the plant (Gorbatsevich et al., 2013). The survival duration of the pathogen *in planta* is very short (<30 h), possibly due to the presence of antibacterial substances of the essential oils in this aromatic herb. Salinity was previously demonstrated to affect the content of essential oils in sweet-basil (Bernstein et al., 2009b) and in other aromatic plants (El-Fadl et al., 1990) as well as oil composition (Tarchoune et al., 2013). Although *S. enterica* strains can tolerate a wide range of salinities (Kang et al., 2007), the induced osmotic pressure might also affect the physiology of the pathogen. It is therefore feasible that persistence of the bacteria in the plant tissues will be affected by exposure of the host plant to saline conditions during cultivation.

Sweet basil (*Ocimum basilicum L*.) is a popular medicinal, culinary and ornamental herb (Roberto and Simon, 2000; Simon, 2000). It is marketed as a dry and fresh herb and is also cultivated for industrial production of essential oils (Dudai et al., 2002). The essential oil, of sweet basil is rich in phenolic compounds, and is used in the pharmaceutical and perfume industries throughout the world

This study was undertaken to evaluate the following hypotheses: (i) Salinity stress affects root internalization of *S. enteric*a serovar Newport into sweet-basil. (ii) *In planta* transport of the human pathogen is reduced under salinity. (iii) Salinity affects survival kinetics of the human pathogen in the leaf tissues of sweet-basil. To test these hypotheses we studied: (1) Effects of plant exposure to salinity on the internalization of *Salmonella* into the plants via the root system, (2) Effect of salinity on the survival of *Salmonella* within leaf tissues. (3) Growth kinetics of *Salmonella* in plant sap from plants cultivated with non-saline or saline stressed plants. (4) Effect of salinity on survival of *Salmonella* in the root growing medium (5) Plant physiological parameters associated with plant response to salinity (salts and mineral accumulation, osmotic potential, relative water content).

## Materials and Methods

### Plant Material and Growing Conditions

The effect of soil salinity on the ability of *Salmonella* cells to enter the roots and translocate to the aerial parts of the plant and survive *in planta* was studied in pot-grown basil plants. Twenty-day-old basil seedling (*O. basilicum*, cv. Perrie) (Dudai et al., 2002) were transferred to 1500 ml black plastic pots, one seedling per pot. Each pot contained 1000 g of water-saturated potting mixture consisting of 40% peat, 40% Coir (coconut fibers), 20% tuff (granulated volcanic ash), and added fertilizers. The pre-mixed fertilizers consisted of a 4-months slow-release Multicot (20:10:20 NPK) 2 kg m^-3^ potting mixture, and 0.5 kg 20:10:15 NPK per m^-3^ of potting mixture as a soluble starter with added microelements. The pots were kept in a naturally ventilated greenhouse, (20°C and 42°C minimum and maximum temperatures, respectively) and were watered daily. The plants were cultivated for 10 weeks, from the seedling stage until flowering.

The salinization treatment was initiated when the plants were 30 days post-germination. The plants were drip-irrigated daily, from day 30 to 60, with non-saline water (control) or water containing 30 mM NaCl (salt-treatment). To prevent over salinization of the growing media the irrigation volume was regulated to allow generation of 25–50% of leachate. Throughout the salinization period the EC (electric conductivity) of the leachate was monitored regularly and did not increase above 3.5 dSm^-1^, i.e., 0.5 EC unit above the EC value of the irrigation solution in the salt treatment.

### Physiological Characteristics of the Plant Tissue

A flow-chart describing the experimental set-up is presented in Supplementary Figure 1. Leaves of 60-day-old plants, were sampled for the analysis of Na, Cl, K, Mg, Ca, osmotic potential, relative water content, antioxidative activity, content of essential oil in the leaves, and root and shoot fresh-weight (FW). Parallel leaves were used for the bacteriological studies in the project. All measurements were conducted with five biological repeats, for plants irrigated with non-saline and saline water. Na and Cl were analyzed as described in Bernstein et al. (2009b). Potassium, Mg, and Ca were analyzed as described in Neves-Piestun and Bernstein (2005). Root and shoot FW, area of individual leaf, and contents of essential oils were determined as described by Bernstein et al. (2009b). Osmotic potential measurements were conducted following Shoresh et al. (2011). Antioxidant activity was measured by a radical scavenging assay using 1, 1-diphenyl-2-picryldrazyl (DPPH) according to Bernstein et al. (2009a) and the antioxidant activity is expressed as chlorogenic acid equivalent. Photosynthesis and stomatal conductance measurements were conducted with a Li-Cor 6400 gas exchange photosynthesis system (Li-Cor, Lincoln, Nebraska, USA) at 400 μmol mol^-1^ CO_2_, 21% (v/v) O_2_, air RH range of 30 ± 1.5%, the temperature was 32 ±0.5°C and photosynthetic active radiation (PAR) of 200 μ m^-2^s^-1^. Measurements were performed after 3 h of light. Relative water content in the plant tissue, in percentage, was calculated as of the equation RWC (%) = 100 × (Wf-Wd)/(Wt-Wd). Wf is fresh weight of the leaf tissue; Wt, is weight of the turgid leaf tissue following hydration in double distilled water for 24 h; and Wd is dry weight of the leaf tissue. Root branching was measured as the averaged number of 2nd order laterals per cm root length in 10 cm long white roots. Data for each replicated plant was averaged from measurements of three roots. The experiment was repeated three times.

### Bacterial Strain and Inoculum Preparation

*Salmonella enterica* serovar Newport (strain 96E01153C-TX) was used throughout the study. This strain was originally isolated from alfalfa seeds associated with an outbreak of salmonellosis (Inami and Moler, 1999). It was used in previous studies (Barak et al., 2002; Charkowski et al., 2002) and was deposited in the ‘California Department of Health Services Microbial Diseases Laboratory Branch’ collection (Friedman et al., 2002). Bacterial cultures were kept as glycerol stocks at -20°C. Before each experiment fresh cultures were grown overnight in Lysogeny broth (LB) broth (Difco, Lawrence, KS, USA) supplemented with nalidixic acid (30 μg ml^-1^) and streptomycin (50 μg ml^-1^) under shaking (150 rpm) at 37°C. Bacteria were washed once by centrifugation (6000 rpm for 5 min at 20°C) and suspended in sterilized distilled water (SDW) to a final concentration of 1 × 10^9^ cfu ml^-1^. Bacterial concentration was determined essentially following Golberg et al. (2011) and Gorbatsevich et al. (2013), except that 10-fold dilutions were plated on xylose lysine deoxycholate (XLD) agar (Difco, Lawrence, KS, USA) plates supplemented with nalidixic acid streptomycin in order to confirm the presence of the inoculated *Salmonella* strain.

### Bacterial Internalization through the Root

Sixty-day-old control and salinized plants, 20 plants per treatment were used for quantification of bacterial internalization through the root. The experiment was repeated three times. In each replicated experiment, the soil in half of the pots was inoculated with *Salmonella*, and bacterial concentration in the shoot was determined 24 h later to allow time for root uptake and *in planta* translocation to the shoot. The concentration of *Salmonella* in the shoot in each experiment was quantifies in 10 independent replicates, i.e., 10 inoculated plants, and 10 non-inoculated plants in each of the non-salinized (control) and the salinity treatments. To minimize contamination of the aerial parts of the plant during inoculation and by direct contact with the contaminated potting mixture, the experimental pots were individually covered with a plastic sheet as is described by Bernstein et al. (2007a). In short, the experimental pots were individually covered above the growing medium with a white, opaque, perforated polypropylene sheet (50 μm thickness; Salina, Nazareth Illit, Israel). The seedlings emerged through a 30-mm-long slit in the polypropylene cover. Ten pinpuncture holes in each cover allowed additional aeration.

Average night and day temperatures in the greenhouse throughout the duration of the experiments were 25.0 ± 0.4 and 27.0 ± 0.6°C, respectively. Pot inoculation was performed by carefully pipetting under the plastic cover 10 ml of bacterial suspension containing 10^9^ CFU ml^-1^ (inoculation treatment) or 10 ml of water (control). Final concentration of bacteria in the soil was 10^6^ CFU g soil^-1^. Twenty-four hours after inoculation of the potting medium ten plants per treatment were aseptically excised, 5 cm above the pot cover. Ten grams of plant tissue was sampled for bacteriological analysis from each replicated pot. For quantification of *Salmonella* in the experimental pots, 1 g of wet potting medium, at 75–80% of field capacity, was sampled from the root growing zone area 24 h after inoculation, in parallel to the collection of plant material for the bacteriological analysis. Bacterial quantification in plant- and soil samples was performed as described below.

### Survival of *Salmonella* in the Leaf

Survival of *Salmonella* in the leaf was evaluated with an experimental system developed to generate consistent loading of bacteria into the plant tissue. The cut edge of the petiole of excised leaves from 60-day-old plants cultivated as described in section 2.4 above, was immersed in 1.5 ml of *Salmonella* suspension in an eppendorf tube containing 1 × 10^9^ CFU ml^-1^. Each tube was sealed with parafilm to prevent contamination of the leaf surface, and to ensure that all water loss is due to uptake by the plant. The leaves were allowed to take up the bacterial suspension or clear water for 18 h at 25°C. Five replicated leaves from each treatment were taken for enumeration of *Salmonella*. The petioles of the remaining leaves were gently washed in water to remove unattached bacteria and the leaves were transferred to new tubes filled with sterilized fresh water. *Salmonella* enumeration was performed at 6, 22, and 28 h after the transfer. The tissue analyzed included the excised leaf tissue, excluding the petiole section which came in contact with the loading suspension. Each experiment was performed with five replicated leaves and the experiment was repeated three times on different days. Leaves selected for the analysis were the largest mature leaves on the plant, from the 8th to 10th node down from the top of the main branch. To confirm the internal localization of the loaded *Salmonella* within the leaf tissue, leaves were disinfected with 1% AgNO_3_ following Franz et al. (2007) and Gorbatsevich et al. (2013). Preliminary testing confirmed that the disinfectant kills the *Salmonella* on the leaf surface. Specifically, no bacteria were detected in leaf prints on LB agar after surface sterilization, demonstrating the efficiency of the surface-disinfection; and no *Salmonella* colonies were detected in leaf prints prior to the surface disinfection, negating the possibility that *Salmonella* reached the aerial parts by external migration. No *Salmonella* were identified also in the clean water after immersion of the loaded leaves, demonstrating that the reduction in bacterial number in the phyllosphere was not a result of their migration to the water (Gorbatsevich et al., 2013). Enumeration of *Salmonella* was performed as described previously (Golberg et al., 2011; Gorbatsevich et al., 2013). Furthermore, no difference in the number of *Salmonella* CFU was detected among leaves prior to and after surface sterilization (data not shown), supporting that the identified cells resided internally.

### Survival of *Salmonella* in Plant Sap *In Vitro*

The effect of salinity on the kinetics of *Salmonella* survival was investigated also *in vitro* in plant sap. The sap was expressed from fresh vegetative tissue of sweet-basil plants irrigated with non-saline vs. saline irrigation water. The experiment was conducted with 10 biological repeats and was repeated three times. The plants were sampled for the analysis 60 days post-germination. For the expression of the sap, fresh basil leaves were ground with a coffee mill pre-sterilized with 70% ethanol. The ground plant material was centrifuged at 10000 rpm for 15 min at room temperature and the obtained sap was filtered through a 0.22 μm membrane for removal of bacteria. One hundred microlitre of the filtered sap was pipeted into a 1.5 ml eppendorf tube and was spiked with 10 μl of the bacterial suspension to give a final concentration of 10^3^ cfu ml^-1^ *Salmonella*. The tubes were incubated at 30°C and the concentration of the bacteria in the sap was followed over 24 h. Bacteriological analysis was conducted as described above.

### Effect of Salinity on the Fate of *Salmonella* in the Potting Medium

The effect of saline irrigation on microbial survival in potting medium was tested in the experimental pots without plants. This allowed to evaluate the sensitivity of *Salmonella* to salinity under the experimental conditions. Fifty 1500 ml black plastic pots, same as those used for plant cultivation, were each filled with 1000 g of the potting medium (Tuff Merom Golan, Merom Golan, Israel). The potting medium was saturated as was previously described (Bernstein et al., 2007a) with water containing a range of NaCl salinities (0, 20, 50, 150, and 500 mM NaCl), 10 pots per salinity level. The experimental pots were individually covered above the soil level as previously described (Bernstein et al., 2007a). Sixteen hours thereafter, five pots per salinity level were each inoculated with 40 ml of suspension containing 10^9^ CFU ml^-1^ *Salmonella* Newport (10^7^ CFU g^-1^ soil). The pots were kept in a greenhouse under the same conditions practiced for plant cultivation. Potting medium from five inoculated and five uninoculated pots for each salinity level was sampled for bacteriological analysis of *Salmonella* and for total aerobic bacterial count, 24 h and 5 days following inoculation. The experiment was repeated three times.

### Bacteriological Analysis of Plant and Soil Samples

Plant tissue sampled for bacteriological analysis from all experiments was first surface sterilized by dipping in 1% AgNO_3_ for 10 s, followed by two washing steps 10 s each in SDW as is described above for the ‘in leaf’ survival experiments. The effectiveness of this method for basil was demonstrated before (Gorbatsevich et al., 2013). The plant tissue was then cut into several pieces (about 1 cm^2^ each), and placed in a pre-weighed plastic stomacher bag (Plastiques gosselin, Borre, France) to determine its fresh weight. Buffered peptone water (BPW; 50 ml) was added to each bag, and the contents were crushed for 1 min by a stomacher (Interscience BagMixer 400, Saint Nom La Bretèche, France). Enumeration of *Salmonella* CFU in the pummeled suspensions was conducted following Gorbatsevich et al. (2013). Since preliminary studies demonstrated that *Salmonella* concentration in the suspension was sometimes below the detection limit (<100 CFU g plant^-1^), the BPW suspension was also further incubated for 24 h at 37°C for pre-enrichment and then used for a qualitative assessment of the presence of *Salmonella* by plating on XLD plates. If no suspected *Salmonella* colonies were observed on the XLD agar after the 24 h pre enrichment, 1 ml of BPW pre-enriched culture was enriched in 9 ml of tetrathionate brilliant green (TBG) and in 9 ml of Rappaport-Vassiliadis soy peptone (RVS) broths (Hy-Labs, Rehovot, Israel). After incubation overnight of TBG (at 37°C) and RVS (at 42°C) the presence of *Salmonella* was determined following Golberg et al. (2011) and Gorbatsevich et al. (2013).

For quantification of *Salmonella* in the potting medium, 5 g of potting medium was taken from each pot and placed in 50-ml tubes, containing 45 ml of BPW. The samples were mixed for 1 min each at maximum speed with a Genie 2 vortex and then incubated on a shaking platform for 1 h at 25°C, to facilitate release of medium-associated bacteria. Determination of *Salmonella* concentration in the potting medium extracts was performed by plating 100 μl of 10-fold serial dilutions on XLD agar following incubation overnight at 37°C. Duplicate plating was performed for each determination. The plates were examined after incubation and typical black colonies were presumptively identified as *Salmonella*. No such colonies were found in any of the control treatments (uninoculated potting medium). To confirm black colonies observed after the plating in either plant or soil samples as *Salmonella*, additional biochemical and serological tests were performed following the U.S. Food and Drug Administration Bacteriological Analytical Manual methods^1^. For biochemical evaluation suspected *Salmonella* cells were grown on TSI (triple sugar iron) and LI (lysine iron) agar (Hy-Labs, Rehovot, Israel). For serological evaluation, somatic (O) antiserum was used (central laboratories of Ministry of health, Jerusalem, Israel). Potting medium samples were also tested for total culturable aerobic bacteria by plating decimal dilutions of the BPW suspension on LB plates and incubating the plates overnight at 37°C. Following the experiments, all the contaminated materials, including plants and potting medium were decontaminated by autoclave before disposal.

### Statistical Analysis

Statistical analysis was carried out with the JMP software package, version 5 (2002, SAS, Cary, NC, USA). Means and standard errors of *Salmonella* CFU in the potting medium and plant tissues, and the total bacterial count in the potting medium were determined. Data were subjected to one-way ANOVA analysis and Tukey honestly significant difference for comparison of means. Most probable number, MPN, was calculated as described by Blodgett (2010), for a ‘single dilution with positive tubes’.

## Results

### Plants Response to Salinity

**FIGURE 1:**
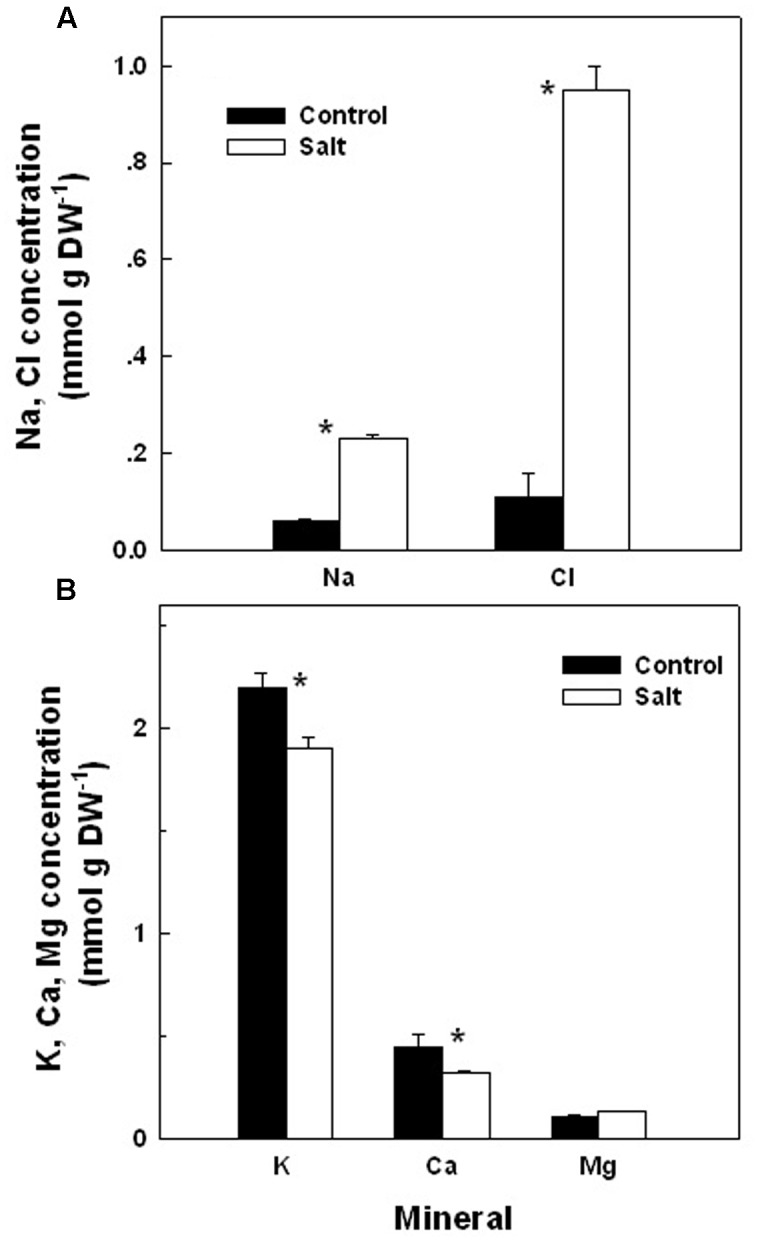
**Effect of irrigation with saline water on the concentrations of the salts Na and Cl (A)**, and the macronutrients K, Ca, and Mg **(B)**, in leaves of plants cultivated under non-saline (Control) and saline irrigation (Salt). The data are means ± SE. Asterisks represent a significant difference between the concentration of a mineral element in control vs. salt plants (*P* < 0.05).

**FIGURE 2:**
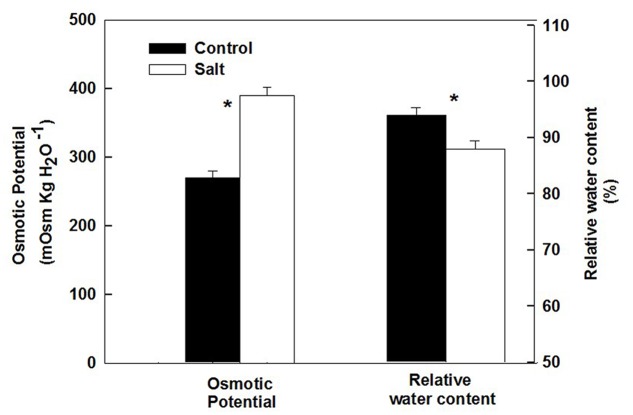
**Effect of irrigation with saline water on osmotic potential and relative water content of leaves from plants cultivated under non-saline (control) and saline irrigation (salt)**. The data are means ± SE. Asterisks represent a significant difference between the control and salt plants (*P* < 0.05).

The basil plants demonstrated a characteristic response of plants to salinity. Growth of the root and the shoot and the area of individual leaves were reduced under salinity (**Table 1**). The decrease in shoot/root biomass ratio represents higher growth sensitivity of the shoot compared to the root to salinity. Salinity induced a change in the nutritional status of the plant tissues: the salinity source ions, Na and Cl, increased about 4- and 9-fold, respectively, under salinization in the leaf tissues (**Figure 1A**) and the concentration of the macronutrients Ca and K were significantly reduced (**Figure 1B**). The osmotic potential of the tissue sap increased by 30%, and the relative water content of the tissue reduced by 6% (**Figure 2**) representing a change in the water status of the plant. Furthermore, CO_2_ fixation and stomatal conductance were reduced by 29 and 26%, respectively; and the % of essential oil content in leaves was increased by 26% (**Table 1**). The increase in antioxidant activity (by 30%) demonstrates a stress-induced change in the oxidative state of the plant tissue (**Table 1**).

**Table 1:** Effect of irrigation with saline water on morphological and physiological characteristics of the plants.

Parameter	Control	Salt	% change
Root FW (g)	15.1 ± 1.3 a	12.4 ± 1.1 b	20.1↓
Shoot/root FW ratio	8 ± 0.2 a	7.2 ± 0.2 b	10↓
Area of individual leaf (cm^2^)	49.2 ± 2.3 a	40.1 ± 1.3 b	19.5↓
Root branching (laterals cm^-1^)	2.5 ± 0.15 b	3.0 ± 0.12 a	20↑
Antioxidant activity (mg gDW^-1^)	56 ± 4.3 b	85 ± 5.2 a	30↑
Photosynthesis (μmol m^-2^ s^-1^)	8.5 ± 1.0 a	6.0 ± 0.6 b	29↓
Stomatal conductance (mol m^-2^ s^-1^)	0.19 ± 0.03 a	0.14 ± 0.02 b	26↓
Essential oil content (% of FW)	0.065 ± 0.007 b	0.082 ± 0.003a	26↑

*‘Control’ represents plants cultivated under irrigation with tap water; ‘salt’ represents plants irrigated with 30 mM NaCl salinity. The data are means (±SE). Means within a row marked by different letters, are significantly different according to Tukey test at α = 0.05. The arrows in the ‘% change’ column represent the direction of the imposed change by salinity, i.e., increase or decrease*.

### Effect of Salinity on the Ability of *Salmonella* to Contaminate Plants via the Roots

The susceptibility of the plants to *Salmonella* penetration via the roots did not vary with exposure of the roots to salinity (**Table 2**). In both salinized and non-salinized plants, no *Salmonella* cells were identified in the above ground plant tissue by direct plating, and the same number of replicates (5 out of 10) were tested positive for *Salmonella* after an enrichment step (**Table 2**). MPN for positive identification in the enrichment tests was 70.7g^-1^ with a 28.9 (low) and 173.7 (high) 95% confidence limit. The concentration of the pathogen in the potting medium, and the total bacterial count, were not affected as well by the saline irrigation (**Table 2**). No *Salmonella* were observed in uninoculated plants and on the surface of disinfected leaves (data not shown) supporting that the identified *Salmonella* was localized to inner tissues of the plants.

**Table 2:** Effect of irrigation with saline water on the internalization of *Salmonella* into plants via the root system.

	Presence of *Salmonella* in aerial plant tissues				Bacterial count in the potting medium (CFU g^-1^)	
	Not inoculated		Inoculated		Total aerobic count	*Salmonella*
Irrigation treatment	Pre-enrichment (CFU g^-1^)	Positive identifications in enrichment tests	Pre-enrichment (CFU g^-1^)	Positive identifications in enrichment tests		
Control	ND	0/10	ND	5/10	7.0 × 10^6^± 5.0 × 10^4^a	8.3 × 10^5^± 2.8 × 10^4^a
Salt	ND	0/10	ND	5/10	7.1 × 10^6^± 4.8 × 10^4^a	7.9 × 10^5^± 3.2 × 10^4^a

*The potting medium was inoculated with 10^*6*^ CFU g^-*1*^ 24 h prior to sampling while at 90% of its ‘field capacity.’ X/10 denotes the number of pots, out of 10 replicates, at which *Salmonella* was identified following enrichment. The data are averages ± SE. Means within each column, followed by different letters, are significantly different according to Tukey test at α = 0.05. ND, not detected (<100 CFU g plant^-*1*^). The detection limit for the enrichment test is <1 CFU/g^-*1*^*.

### Effect of Salinity on the Survival of *Salmonella* in Leaves

**FIGURE 3:**
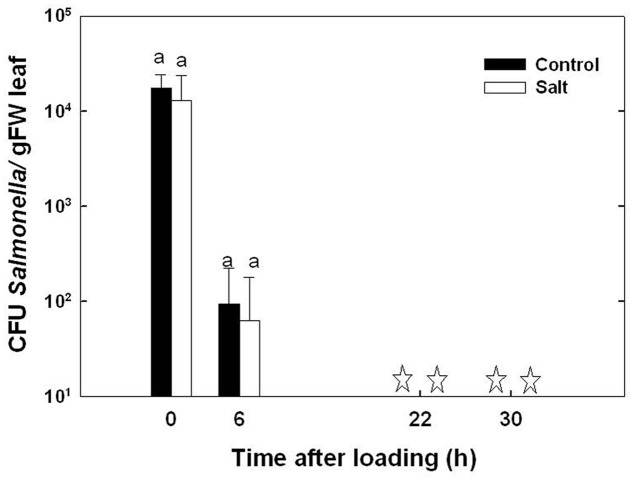
**Effect of irrigation with saline water on the survival of *Salmonella* within the leaf**. The presence of *Salmonella* in the leaves was determined at the end of the pathogen loading period (time 0), and 6, 22, and 30 h thereafter. ‘Control’ represents leaves from plants cultivated under irrigation with tap water; ‘salt’ represents leaves from plants irrigated with 30 mM NaCl salinity. The data are means (±SE). Means in a single sampling day, marked by same letters, are not significantly different according to Tukey test at α = 0.05. Asterisks represent that no bacteria (< 1 CFU/g^-1^) were detected in the leaves even after an enrichment test.

The survival duration of *Salmonella* in the basil phyllosphere was investigated in leaves from plants cultivated under non-stressed and salt-stressed conditions. A considerable reduction in *Salmonella* counts in the leaf tissue was apparent already 6 h after the end of the inoculation period, and no *Salmonella* was detected 16 and 24 h thereafter (**Figure 3**). The rate of decrease in *Salmonella* CFU was not significantly different in leaves from control and salt-stressed plants, ranging 2.9 × 10^3^ - 2.1 × 10^3^ CFU g^-1^ day^-1^ at the first 6 h following inoculation (*p* = 0.554). The lack of effect of the salinity exposure on *Salmonella* occurred despite the changes in chemical and physical parameters in the plant.

### Kinetics of *Salmonella* Growth in Sap Expressed from Control and Salinized Plants

**FIGURE 4:**
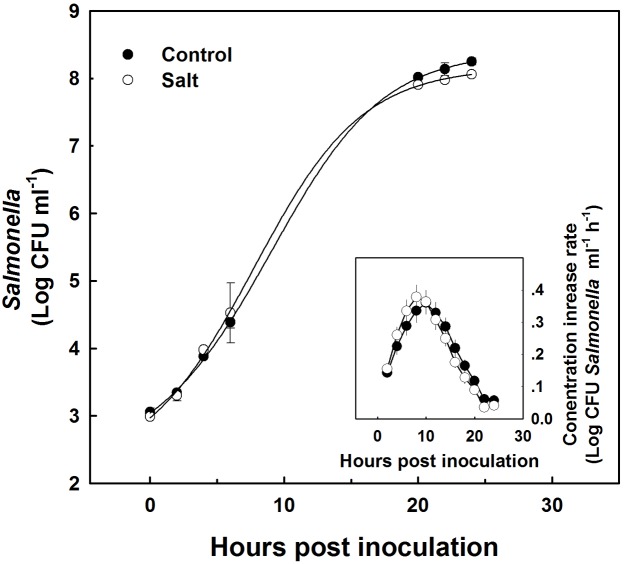
**Growth kinetics of *Salmonella* in plant sap expressed from shoots of sweet basil plants cultivated with non-saline (control) or saline irrigation (salt)**. Changes in *Salmonella* concentration (main figure), and rate of concentration change (insert) over time following spiking of the sap with 10^3^ CFU ml^-1^ *Salmonella*. In the main figure, time 0 present results immediately following sap inoculation; and the lines represent a sigmoidal curve-fit to the data. The data are means ± SE. In the insert, the averaged rates and SE were calculated from the sigmoidal curve-fit data for the individual biological repeats. The difference in concentration and rate between the control and the salt treatment was not statistically different (*P* < 0.05) for any of the sampling times.

**FIGURE 5:**
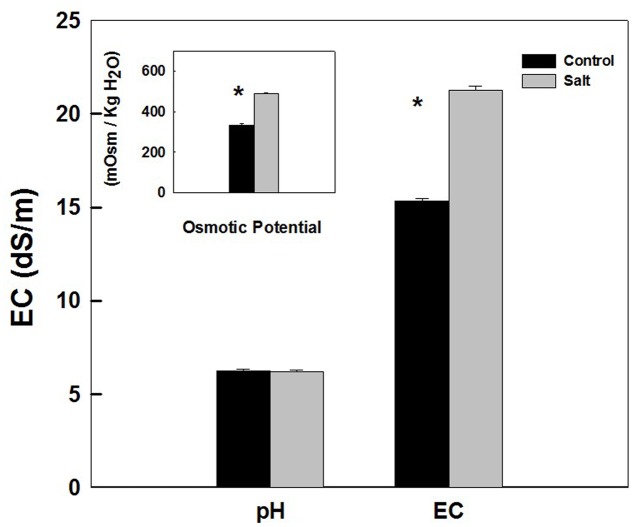
**pH, electrical conductivity (EC), and osmotic potential of the plant sap used for the *in vitro* experiments described in **Figure 4****. The sap was expressed from shoots of sweet basil cultivated under non-saline (Control) and saline irrigation (Salt). The data are means ± SE. Asterisks represent a significant difference between the control and salt plants (*P* < 0.05).

Sap extracted from leaves of control as well as salt-stressed plants supported growth of the *Salmonella* population *in vitro* (**Figure 4**). The kinetics of bacterial population increase was similar in sap from plants cultivated under control and salt conditions, demonstrating a sigmoidal curve with leveling off at 20 h after inoculation (**Figure 4**). The rate of growth was similar under control and salt conditions (**Figure 4** insert). No statistical difference was found between the control and salt treatment in the concentration (**Figure 4**) or the concentration increase rate (**Figure 4** insert) at any of the time points analyzed (*p*-values ranged: 0.857–0.098). The lack of changes in the kinetics of bacterial growth *in vitro* occurred despite the physical differences between the sap of the two treatments. Both electrical conductivity (EC) level and the osmotic potential of the sap significantly increased under salinization, while no changes in pH level were apparent (**Figure 5**).

### Effect of Saline Irrigation Water on Microbial Survival in the Potting Medium

**FIGURE 6:**
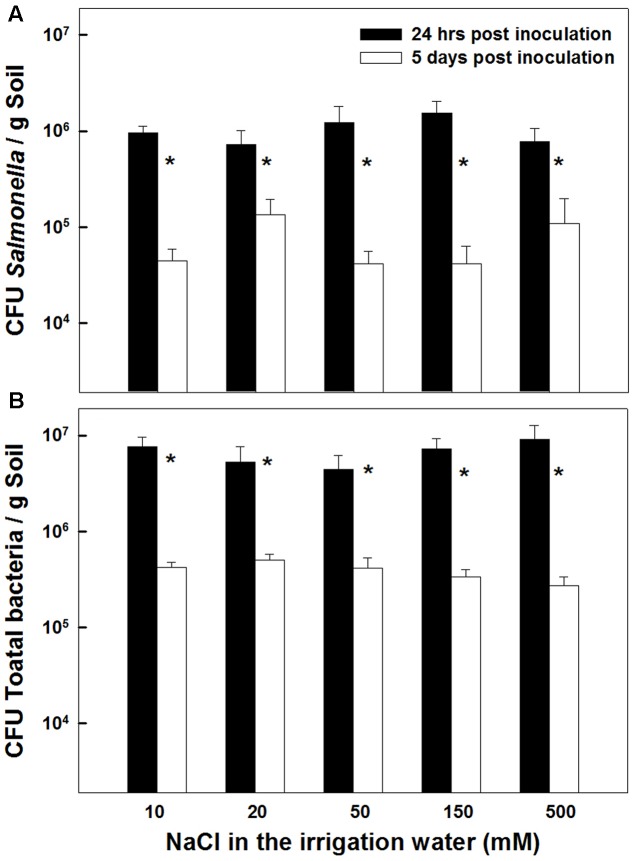
**Effect of irrigation with saline water on survival of *Salmonella* (A)**, and total aerobic bacteria **(B)**, in the potting medium 24 h, and 5 days following inoculation. Data are averages ± SE. The soil was inoculated with 10^7^ CFU *Salmonella* g soil^-1^. Asterisks represent significant difference of the measurement on the 1st vs. 5th day after inoculation (*P* < 0.05) at any salinity level.

The concentrations of *Salmonella* and total aerobic bacteria in the potting medium were not affected by the salinity level applied (0–500 mM NaCl) (**Figures 6A,B**). In both measurements conducted, 24 h and 5 days after inoculation, no statistical differences were recorded in *Salmonella* concentrations or total aerobic bacterial counts throughout the salinity range tested (*P* = 0.2132 and 0.1875 for 24 h and 5 days after inoculation, respectively). However, the concentrations of *salmonella* as well as the total bacterial count in the potting medium were reduced over time, i.e., between the first and second measurement. A 1–1.5 log CFUg^-1^ reduction was recorded between the first and second measurement (**Figures 6A,B**). This similarity in the extent of concentration decline throughout the salinity concentration range, suggests that the decrease observed over time in the total bacterial count, reflects die-off of the *Salmonella* population. It also suggests that the total heterotrophic populations were not very high in the potting soil.

## Discussion

### Effect of Salinity on Shoot Contamination by Root-Internalized *Salmonella*

The presence of root-internalized *Salmonella* in the shoot is an integrated result of root internalization, transport to the shoot and *in planta* persistence. Salinity induces a range of changes in the plant, which may singularly or collectively affect each of these processes and hence the subsequent internal contamination of the shoot by human pathogens present in the soil. The results of this study demonstrate that internal shoot contamination was not affected by saline irrigation (**Table 2**), suggesting that plant’s susceptibility to root internalization, transport to the shoot and persistence were not compromised by the salinity-induced physiological or morphological changes.

The natural ruptures created by the emergence of lateral roots from the pericycle through the cortex and epidermis are an accepted possibility for entrance ports of pathogens into roots (Lugtenberg et al., 2001; Jablasone et al., 2005; Hirneisen et al., 2012). The increase in root branching observed under salinity (**Table 1**) increased the number of entry points for the bacteria and hence the potential for root internalization. Additionally, the reduction in shoot/root ratio under salinity (**Table 1**) which indicates that compared to the control plants, under salinity a larger root mass supported internalization into a unit mass of leaves, represents as well increased potential for internalization into a unit leaf mass. On the other hand, the smaller root system that developed under salinity (**Table 1**; Bernstein et al., 2009b) restricted the cumulative number of laterals in the root, and thereby the opportunity for bacterial root internalization. The reduced stomatal conductance and hence the potential for transpiration rate under salinity (**Table 2**) lowers the volume of ascending sap that reaches the leaf and is therefore expected as well to have a restrictive effect on the transport of soil-contaminating bacteria to the shoot. The lack of salinity effects on the incidence of root-internalized bacteria in the shoot (**Table 2**) could result from integration and balancing out of these opposing affects (**Table 1**). Zhang et al. (2009) reported that heat and drought stress did not affect the susceptibility of plants (romaine and iceberg lettuce) to contamination by root uptake of *E. coli* O157:H7, and DiCaprio et al. (2015) reported that drought stress but not flooding stress affected the rates of internalization and dissemination of Human norovirus into lettuce.

### Effect of Salinity on the Survival of *Salmonella In Planta*

Following root internalization the enteric pathogens are exposed to the internal environment of the plant. Numerous plant characteristics, including morphology, water content, phosphate concentration, amounts of bacterial inhibitory phenolics, leaf thickness and endophytic bacteria were shown to affect bacterial populations in the phyllosphere (Thompson et al., 1993; Yadav et al., 2005; Gu et al., 2013; Wiedemann et al., 2015). The prevalence of enteropathogens beneath the epidermis of leaves was found to vary between plant species (*Salmonella* Typhimurium: Golberg et al., 2011), and between abaxial and adaxial internalization into leaves (*E. coli* O157:H7: Erickson et al., 2010). Despite the differences in chemical and physical parameters in leaves of the salt-stressed and non-stressed plants (**Table 1**) the rate of decrease in *Salmonella* counts was similar under control and saline conditions (2905±350 and 2130±350 CFU h^-1^ for the control and salinity, respectively, at the first 6 h after loading, **Figure 3**). The lack of effect of saline-irrigation on the survival of *Salmonella* in the tissue suggests that the levels reached in the leaves (0.23 and 0.95 μ g DW^-1^ of Na and Cl, respectively) were not toxic for the bacteria, as could be judged also by the salt-challenge experiments (**Figure 6**). Tolerance of *Salmonella* to higher salinities, was also documented (1M NaCl, Kang et al., 2007). Similarly, the reduction in concentration of the macronutrients K and Ca under saline irrigation, did not affect the persistence of *Salmonella* in the plant, suggesting that the reduced levels were within the range sufficient for survival or adaptation. Intracellular K is known to involve in the osmo-protection response and regulation of turgor in bacteria including enteric bacteria (Booth and Higgins, 1990; Prince and Villarejo, 1990; Meury and Kohiyama, 1991). The salinity-induced changes in two parameters, (e.g., 30% increase of osmotic potential of the leaf sap, and 6% reduction of the relative water content of the leaf tissue) demonstrate altered water relations in the plant (**Figure 2**). The level of osmotic potential reached under saline irrigation, about 380 mOsm H_2_O^-1^, is well within the tolerance level of *Salmonella* justifying the lack of effects on bacterial survival.

*Salmonella* was able to survive only very short durations within the leaf (<22 h). Sweet-basil is an aromatic plant which produces essential oils that exhibit antibacterial activity (Bagamboula et al., 2004). Essential oils are known to be active against a wide range of microorganisms including food-borne pathogens (Oussalah et al., 2006). The antimicrobial activity of the essential-oils is assigned only to a small number of the oil constituents (Bakkali et al., 2008). Since environmental conditions such as salinity affect concentrations of only part of the oil components (Dudai, 2005), the increase in essential-oil content under salinity (**Table 1**) does not necessarily imply higher concentrations of active antibacterial components and a more pronounced antibacterial activity. The lack of salinity effects on bacterial fitness (**Figure 3**), despite the increased oil content (**Table 1**), may result from stress effects on the composition of the essential oil (El-Fadl et al., 1990). The essential oils are formed in specialized glands on the plant surfaces and are not found in large quantities within the plant tissues. Whether residual essential oils dissipating to inner plant tissues or to the leaf surface between the oil glands can limit *Salmonella* survival in the phyllosphere is still to be determined.

Exposure to abiotic stresses such as salinity is known to influence the plants defense system to microorganisms by priming an induced resistance system (Beckers and Conrath, 2007; Balmer et al., 2015; Ramegowda and Senthil-Kumar, 2015). It is accepted today that there is a connection between biotic and abiotic stresses in plants at the molecular level (Conrath et al., 2002; Borges et al., 2014; Foyer et al., 2016), and that priming enable cross-talk between defense mechanisms against pathogens and the response to abiotic stresses (Maleck and Dietrich, 1999; Waller et al., 2005; Ton et al., 2009; Foyer et al., 2016). Inorganic salts such as Na and Cl, which accumulated under salinity in the basil plants (**Figure 1**), are among the elicitors of phytoalexins (e.g., plant antimicrobial compounds) accumulation (reviewed in Kuc, 1995). The sustained transport and viability of *Salmonella* under saline irrigation suggest that priming or systemic resistance were not induced by salinity or by the presence of *Salmonella*, or that it did not affect the pathogen.

### Kinetics of *Salmonella* Culture Growth in Plant Sap *In Vitro*

*In vitro* experiments with sweet-basil leaf sap, spiked with *S. enterica* serovar Newport, demonstrated similar growth rates of the bacteria in sap expressed from plants irrigated with non-saline, or saline irrigation (**Figure 4**). This is in spite of the higher levels of osmotic potential and EC imposed by the salt-stress. Unlike the leaf phyllosphere (**Figure 2**) the sap expressed from the leaf was able to support growth of the pathogen *in vitro* (**Figure 4**). The micro-environments to which the pathogen is exposed to inside the leaf tissue, vary from the conditions present in the sap expressed from the leaf. For example, in the sap, the pathogen may be exposed to a wider selection of carbon and nitrogen sources otherwise compartmentalized in specific organelles or tissues. While restriction of available nutrients within the leaf might inhibit bacterial growth, the rapid clearance of *Salmonella* in the leaf may perhaps be attributed to the presence of an active innate immunity in the intact tissue compared to the sap. Moreover, essential oils normally found in specialized glands on the leaf surface when incorporated to the sap are much diluted and serve as a food source for the bacteria rather than diminish it. Furthermore, competition with plant natural endophytic microflora, either bacteria or fungi may affect fitness of *Salmonella* inside the plant (Klerks et al., 2007a) but not in the plant sap that was filtered for removal of bacteria. The results for culture-growth kinetics of the *in vitro* experiments complement the results for *in planta* die-off rates that both demonstrate a lack of salinity effects.

### Effect of Salinity on Fate of *Salmonella* in the Potting Medium

It is well documented that a range of human pathogens, including *Salmonella*, can survive extended periods of time in soils (Santamaria and Toranzos, 2003; Gerba and Smith, 2005; Bernstein et al., 2007a; Liu et al., 2010; Jacobsen and Bech, 2012). Two to four months survival periods for enteric bacteria in soil were reported in a review by Gerba et al. (1975), and survival duration up to 29 weeks (Islam et al., 2004) was reported for *Salmonella*. It is therefore not surprising that 5 days following inoculation, a considerable number of viable *Salmonella* cells were detected in the potting medium (**Figure 1A**).

*Salmonella* species are well adapt for survival in diverse environments. However, exposure to a range of environmental stresses, such as nutrient deprivation, pH extremes, oxidative stress, salinity and heat shock, can affect their persistence and virulence (Archer, 1996; Gerba and Smith, 2005). Entero-pathogens encounter osmotic-stress frequently in the host gut, and consequently have evolved an array of stress-adaption mechanisms, that allow them to grow at widely different external osmotic pressures (Brandl, 2006). Result of the present study, which display a tolerance of *Salmonella* to a wide range of irrigation-water salinities (**Figure 6**), demonstrate that under conventional saline irrigation setups utilized for crop production (<500 mM NaCl) as well as conditions applied in the present project, the bacteria is able to persist in the soil.

For practical implications, four issues should be considered. First, the presence of plants in the soil and the soil type may affect bacterial persistence (Islam et al., 2004; Bernstein et al., 2008). Second, the concentration of the pathogen applied to the potting medium in the present study was higher than is expected for sporadically contaminated irrigation water. Pathogen survival in soils may be affected by the initial population size as well as by competition with indigenous populations in the soil, which seems lower in the potting mixture compared to soil-based systems. Third, bacteria response to salinity may be affected by the duration of exposure to the stress. Fourth, the behavior of *Salmonella* may vary in the potting medium used in this study and in various soils, as discussed above. Therefore, more studies are needed before extrapolating bacterial response to salinity in soils and for longer durations then evaluated here. Additionally, bacterial internalization through the root is known to vary with growing conditions, and in most studies involving soil-grown crops, internalization was sporadic and at low levels. It is therefore considered that in undamaged plants, the risk of produce contamination by root internalized bacterial pathogens from contaminated soil may be low (Warriner and Namvar, 2010; Hirneisen et al., 2012; Orlofsky et al., 2016).

## Conclusion

The lack of effect of irrigation with saline water on contamination of the shoot with *Salmonella* suggests that the susceptibility to root internalization, transport to the shoot, and *in planta* persistence were not compromised. Our results, favor the notion that exposure to salinity does not compromise root penetration and/or *in planta* transport and survival of enteric pathogenic within the plant.

## Author Contributions

NB developed the project, analyzed the results, and wrote the manuscript. EG conducted the experiments, SS regulated the microbiological analyses, ND regulated the essential oils analyses.

## Conflict of Interest Statement

The authors declare that the research was conducted in the absence of any commercial or financial relationships that could be construed as a potential conflict of interest.

## Footnotes

1 https://www.fda.gov/food/foodscienceresearch/laboratorymethods/ucm070149.htm
