# Supplementary material for: Salinity Stress Does Not Affect Root Uptake, Dissemination and Persistence of Salmonella in Sweet-basil (Ocimum basilicum)
Source: Front Plant Sci. 2017 May 2;8:675. doi: 10.3389/fpls.2017.00675 (PMC5411819; doi:10.3389/fpls.2017.00675)
Supplement: Supplementary file 1 [file Image_1.PDF]

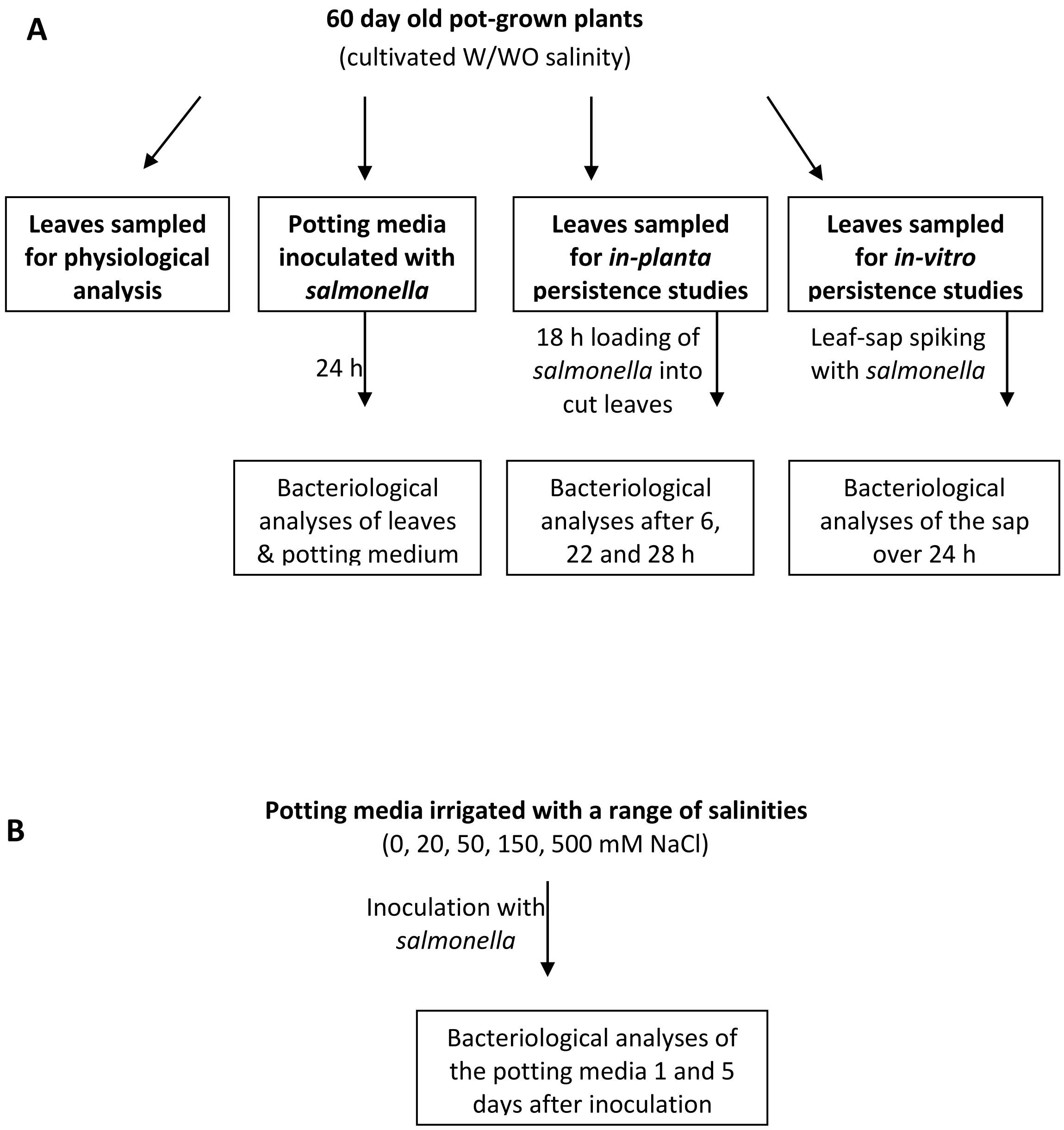

<u>Fig 1 (supplement)</u>: Flow-charts of the experimental set up. A. Plant experiments. B. Potting-media experiment.
